# Optical response of heterogeneous polymer layers containing silver nanostructures

**DOI:** 10.3762/bjnano.8.108

**Published:** 2017-05-16

**Authors:** Miriam Carlberg, Florent Pourcin, Olivier Margeat, Judikaël Le Rouzo, Gérard Berginc, Rose-Marie Sauvage, Jörg Ackermann, Ludovic Escoubas

**Affiliations:** 1 Aix Marseille Univ, Université de Toulon, CNRS, IM2NP, Marseille, France; 2 Aix Marseille Univ, CNRS, CINaM, Marseille, France; 3 Thales Optronics, Elancourt, France; 4 DGA/DS/MRIS, 75015 Paris, France

**Keywords:** nanoprisms, nanospheres, plasmonic nanoparticles, spectroscopic ellipsometry, thin film layers

## Abstract

This work is focused on the study of the optical properties of silver nanostructures embedded in a polymer host matrix. The introduction of silver nanostructures in polymer thin films is assumed to result in layers having adaptable optical properties. Thin film layers with inclusions of differently shaped nanoparticles, such as nanospheres and nanoprisms, and of different sizes, are optically characterized. The nanoparticles are produced by a simple chemical synthesis at room temperature in water. The plasmonic resonance peaks of the different colloidal solutions range from 390 to 1300 nm. The non-absorbing, transparent polymer matrix poly(vinylpyrrolidone) (PVP) was chosen because of its suitable optical and chemical properties. The optical studies of the layers include spectrophotometry and spectroscopic ellipsometry measurements, which provide information about the reflection, transmission, absorption of the material as well as the complex optical indices, *n* and *k*. Finite difference time domain simulations of nanoparticles in thin film layers allow the visualization of the nanoparticle interactions or the electric field enhancement on and around the nanoparticles to complete the optical characterization. A simple analysis method is proposed to obtain the complex refractive index of nanospheres and nanoprisms in a polymer matrix.

## Introduction

Noble metal nanoparticles (NPs) are of considerable interest in various domains, ranging from chemistry to medicine and light filtering [1–6]. Silver NPs are especially challenging because of the possibility to control the phenomenon of light–matter interaction in the visible wavelength range. The optical properties of these metallic NPs are induced by localized surface plasmon resonances, which are size, shape, material and environment dependent [7]. At the localized surface plasmon resonance wavelengths, the conduction electrons of the NPs oscillate coherently, which induces an electric field enhancement. The incoming light is either absorbed or scattered by the NPs [8]. The absorption and scattering are commonly referred to as optical extinction. Single NPs are widely studied under different characterization techniques and computer modeling, such as Mie theory for spherical NPs. The absorption dominates the extinction for small radii and scattering dominates for larger radii. For silver, the threshold radius is 20 nm [9].

Given these properties, the optical properties of the thin films can be engineered by embedding specifically designed NPs. For example, by choosing silver NPs, the thin film layers will absorb in the visible wavelength range. This leads to applications of plasmonic thin film layers for photodetectors [10], photovoltaics [6,11] or nonreflective coatings [12–14].

In this works, silver NPs were chosen for their high electric field enhancement in the visible wavelength range [15]. Progress in the chemistry of NP synthesis allowed us to choose a chemical process adapted to our study that produces NPs of different shapes and sizes. Among different NP production methods, chemical synthesis is easy to implement and produces NPs at low cost. Standard physical vapor deposition methods require high energy sources, such as lasers [16], whereas chemical synthesis produces, among other shapes, nanospheres and nanoprisms of different sizes in water at room temperature. Taking advantage of the size and shape versatility of this chemical synthesis, we aim to control the absorption of thin film layers by embedding different NPs.

When included in polymer thin film layers (such as poly(vinylpyrrolidone) (PVP)), the plasmon resonance wavelengths of the NPs are red-shifted with respect to the resonance wavelengths in water. This environment dependence can easily be understood by considering the variation of the absorption cross-section, σ_abs_, of a spherical NP according to the Mie theory:

[1]
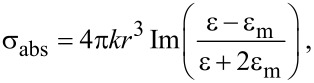


where *k* is the wave vector, ε is the complex dielectric function of the NP and ε_m_ the complex dielectric function of the surrounding medium. Furthermore, collective resonances can alter the optical properties of these NPs and further redshift the absorption peaks.

In the following, we present studies of thin film layers with differently shaped and sized silver NP inclusions. The NPs are obtained by a facile water-based chemical synthesis at room temperature [17–18]. Depending on the chosen reagents, different shapes are achieved. To cover the whole visible wavelength range, we synthesized nanospheres and nanoprisms as their colloidal solutions absorb from 390 up to 1300 nm. The nature of the absorption peak was determined through spectrophotometer measurements coupled with computer simulations and transmission electron microscopy (TEM) imaging. Spectrophotometer and spectroscopic ellipsometry measurements of the PVP host matrix validated its non-absorbing and transparent properties. Spectroscopic ellipsometry measurements of the heterogeneous layers of nanospheres and nanoprisms embedded in the host matrix were fitted by the mathematical addition of the Cauchy law and the single Lorentz law centered at the dipolar resonance wavelength of the nanoparticles. Spectroscopic ellipsometry has been previously performed on nanoparticles embedded in dielectrics [19–20], but the NPs were primarily spherical or ellipsoidal. We propose here a simple analysis of nanoprisms based on the analysis of nanospheres in PVP.

## Results and Discussion

### Characterization of the colloidal nanoparticle solution

**Figure 1:**
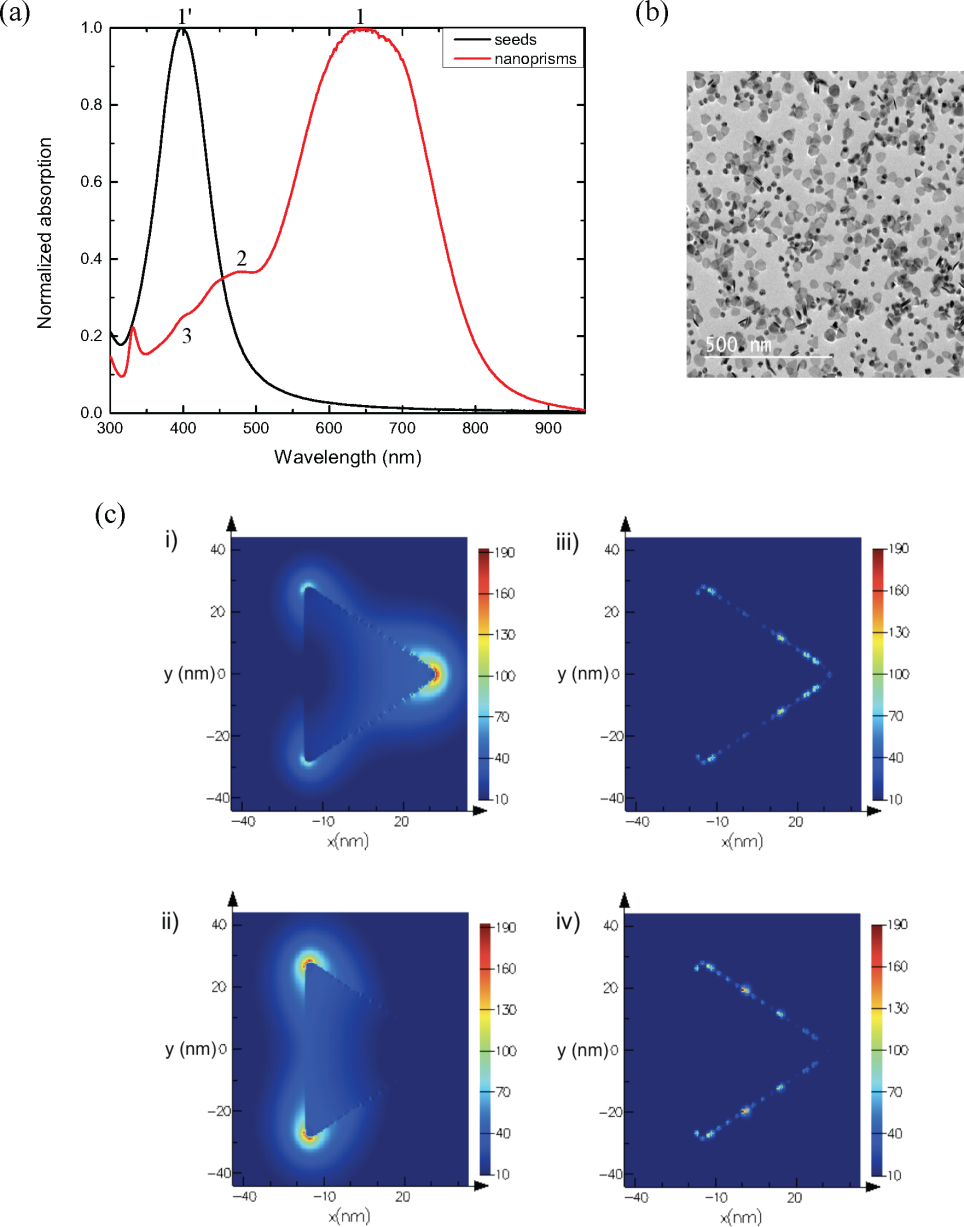
a) Normalized absorption of nanosphere and nanoprism solutions. b) TEM image of the synthetized nanoprisms before centrifugation and c) visualization of the electric field enhancement normalized to the incident electric field in the nanoprism for wavelength of peak 1 under *z*-oriented (i) p-polarized light and (ii) s-polarized light, and for the wavelength of peak 2 under (iii) p-polarized light and (iv) s-polarized light.

The NPs are synthesized as described below in the Experimental section. As synthesized, the nanospheres and nanoprisms were at first dispersed in water. The growth of the nanoprisms was fulfilled in two steps: first spherical seeds with specific crystallographic defects were produced, and second, the growth took place on these defects to form nanoprisms [18,21–22]. The absorption of the colloidal solutions of the first and second step, as seeds and prisms (Figure 1a), shows the characteristic plasmonic absorption peaks. The peaks 1 and 1’ were identified as the dipolar resonance of the nanoprisms and the nanospheres, respectively. The dipolar resonance of the nanoprisms induced absorption between 540–750 nm. The width of this absorption was not due to the distribution in size of the NPs, but rather to the random orientation of the NPs in the solution. The nanoprisms, as synthetized, are visualized in TEM images (Figure 1b). The residual seeds are removed by successive centrifugation steps. Furthermore, finite difference time domain (FDTD) simulations show that the maximum electric field enhancement (Figure 1c) for p- and s-polarized light does not occur at the same wavelength. For a single nanoprism (50 nm edge size and 10 nm thickness) in water, the maximum electric field enhancement for p-polarized light, corresponding to the dipolar resonance wavelength, occurs at 625 nm, whereas the maximum electric field enhancement for s-polarized light occurs at 619 nm.

This absorption peak can be tailored to any wavelength desired in the visible wavelength range by changing the size of the nanoprisms. This is simply done by changing the quantity of seeds added during the second step of the nanoprism synthesis. The spherical seeds absorbed light between 350–430 nm. The nature of the resonances was verified by computer calculations using the Mie theory for the spherical seeds and FDTD for the non-spherical particles. The maximum electric field enhancement for p- and s-polarized light for the dipolar and quadrupolar exaltations are pictured on Figure 1c. Peak 2 was identified as the quadrupolar resonance peak of the nanoprisms. The absorption peak 3 was assumed to be due to the dipolar resonance of the residual seeds in the nanoprism solution from the absorption spectrum. TEM images of the NPs in solution confirmed that residual spheres are present. These different plasmonic resonance peaks presented in the absorption spectrum of the NPs in solution resulted mathematically in the addition of Lorentz functions centered at the different energies.

In summary, silver NPs that absorb over the entire visible wavelength range were synthesized, and the nature of the absorption peak of the colloidal solution was determined. Additionally, the optical response in thin film layers was studied.

### Spectroscopic ellipsometry

Spectroscopic ellipsometry is a powerful technique to determine the effective optical indices (real and imaginary parts) of thin film layers or the thickness of known materials. Indeed, the thickness and the optical indices of the thin film layer are coupled parameters and cannot be controlled simultaneously. To obtain the real and imaginary part of the optical index of the thin films, variable angle spectroscopic ellipsometry (VASE) measurements were performed with a Semilab rotating compensator ellipsometer. The incident beam was focused on a micrometer sized spot diameter of the sample (about 100 μm). We measured the polarization change of light upon reflection on a sample in the visible wavelength range at which the silver NP absorb: 350–950 nm at incident angles of 65°, 70°, 71°, 72°, 73°, 74° and 75°. This polarization change depended on the amplitude and phase variations of the electric fields for p- and s-polarization. More details on spectroscopic ellipsometry can be found in textbooks [23–24].

For data analysis, the films were considered as an effective medium having properties including those both of the NPs and the polymer, as has been previously reported [19–20,25–26]. The optical modeling was developed using SEA (WinElli3) software. This software uses the Levenberg−Marquardt algorithm [27] to minimize the mean squared error (MSE) between the measured and calculated ellipsometric data, Ψ and Δ. To verify the validity of our mathematical model, transfer matrix method (TMM) calculations of the reflectance were compared with reflectance measurements carried out on a spectrophotometer (Lambda 950, PerkinElmer).

It should be noted that the complex refractive index 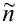 is 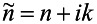, where *n* is the frequency-dependent real refractive index, and *k* is the frequency-dependent extinction coefficient.

### PVP host matrix

To maximize the influence of the nanospheres and nanoprisms on the light interaction, the NP host matrix must be a transparent, non-absorbing polymer in the visible wavelength range. Furthermore, the matrix should be a mild environment for the NPs and help the adherence of the NPs to the substrate [28]. Attempts to deposit the nanoparticles directly on the substrates did not succeed as the optical properties of the measured substrates did not show any nanoparticle optical signature. PVP is commonly used to stabilize silver NPs [29–30]. PVP is also a flexible polymer [31], thus the integration of plasmonic properties into this flexible matrix could be interesting for flexible devices.

First, the polymer alone was studied. To obtain a complete understanding of the host matrix, PVP of different molar weights, 40,000 g·mol^−1^ and 55,000 g·mol^−1^, were deposited in thin film layers. In order to determine the optical indices of the films as a function of the molar weight, a dispersion model based on the Cauchy mathematical law was used for each sample:

[2]
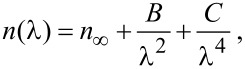


where *n* is the refractive index varying with the wavelength, *n*_∞_ is the refractive index at infinite energy, and *B* and *C* are two constants. The extinction coefficient of the host matrix is zero in the visible wavelength range, i.e., the absorption of the heterogeneous thin films will be only due to plasmonic effects.

**Figure 2:**
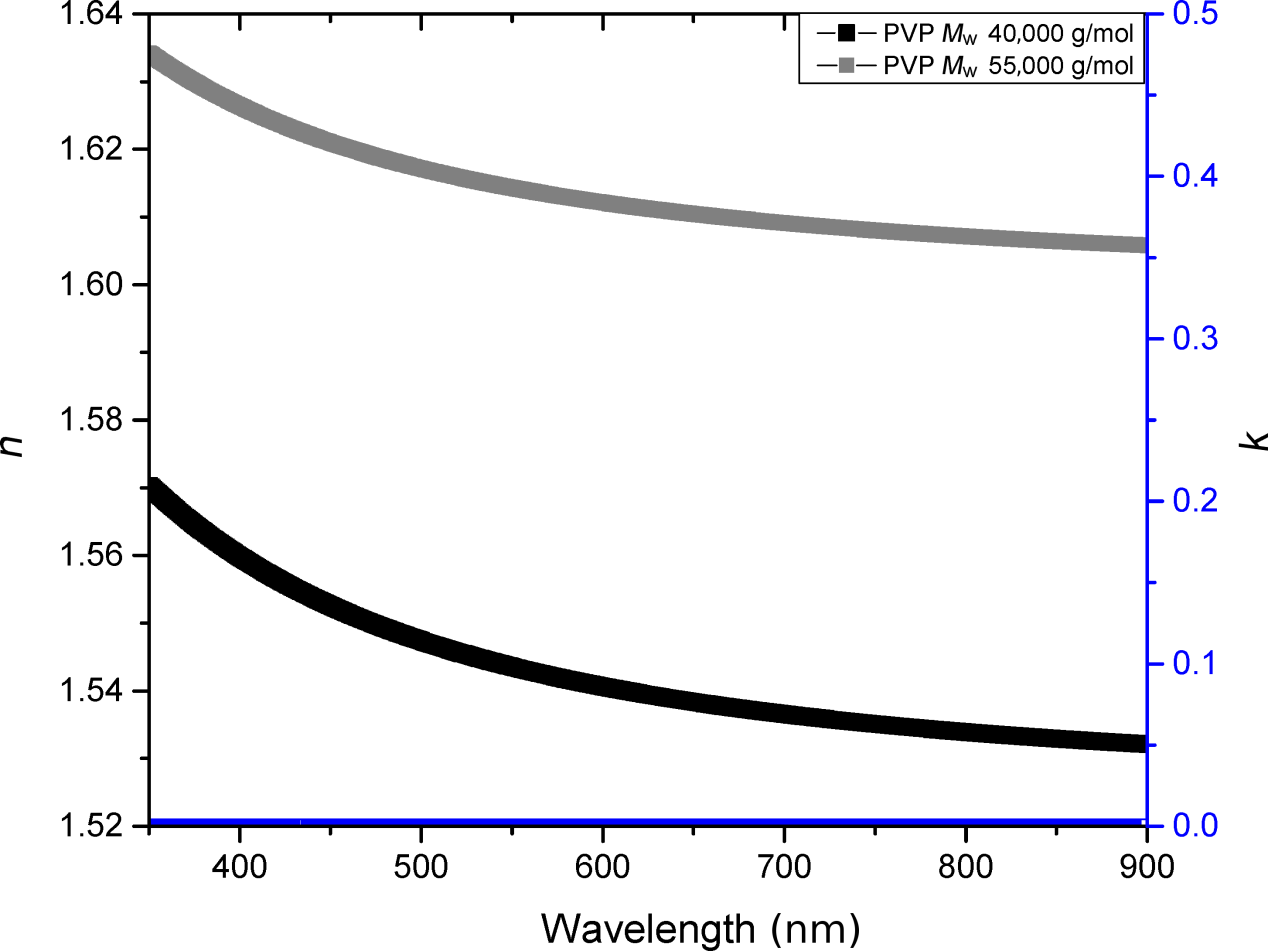
Real and imaginary optical indices of PVP of 40,000 and 55,000 g·mol^−1^ average molar weight, fitted by a non-absorbing Cauchy law.

The parameters for the Cauchy law are listed in Table 1. The measured refractive indices (Figure 2) indicate a dependence on the molar weight. With increasing molar weight, the material fraction in the layer changes, leading to a higher refractive index. The choice of the molar weight therefore has importance when considering the optical properties of the thin film layers.

**Table 1:** Fitting parameters of spectroscopic ellipsometry measurements for different structures.

Sample	Laws	Cauchy parameters	Lorentz parameters

PVP 40,000 g·mol^−1^	Cauchy	*B* = 0.0053 μm^2^, *C* = 0 μm^4^	
PVP 55,000 g·mol^−1^	Cauchy	*B* = 0.00709 μm^2^, *C* = 0 μm^4^	
PVP 55,000 g·mol^−1^ + 20 nm nanospheres	Cauchy + 1 Lorentz	*B* = 0.00544 μm^2^, *C* = 0 μm^4^, *n*_∞_ = 1.57	*f* = 0.005, *E*_0_ = 3.09 eV, Γ = 0.41 eV, ε_∞_ = 0.0002
PVP 55,000 g·mol^−1^ + 25 nm nanoprisms	Cauchy + 1 Lorentz	*B* = 0.00502 μm^2^, *C* = 0 μm^4^, *n*_∞_ = 1.56	*f* = 0.003, *E*_0_ = 1.9 eV, Γ = 0.32 eV, ε_∞_ = 0.0066

In the following, the PVP of molar weight of 55,000 g·mol^−1^ was be chosen for experimental reasons. Taken together, the host matrix was characterized by spectroscopic ellipsometry and fitted by a non-absorbing Cauchy law. The absorption of the thin film layer was derived from the presence of the silver NPs.

### Heterogeneous thin film layers

By embedding NPs into the non-absorbing polymer host matrix, the absorption of light was induced at specific plasmonic wavelength resonances. In order to determine the optical indices of the films containing NPs, the thin film layer was visualized as an effective medium [32–36]. A dispersion model based on a non-absorbing Cauchy mathematical law and one or more Lorentz mathematical laws was used. The Lorentz oscillator model is derived from the classical theory of interaction between light and matter [27]. As it describes the dipolar frequency-dependent polarization due to bound charges, it represents well the plasmonic behavior of metal NPs. The Lorentz model influences both the real and imaginary part of the dielectric function as

[3]
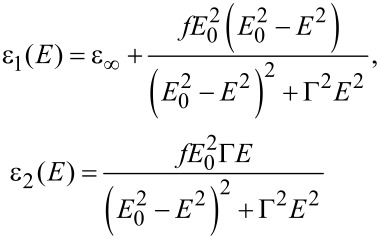


where *f* is the oscillator strength, *E*_0_ (in eV) is the resonant energy of the oscillator, Γ (in eV) is the broadening of the oscillator and ε_∞_ is the high energy dielectric constant.

We focused on the plasmonic properties of the silver NPs in the visible wavelength range. Therefore, the Lorentz model was sufficient to account for the optical properties of the NPs, and the electronic intraband transitions occurring in the UV [27] were not considered in this study. The resonant energy and the broadening width of the oscillator were deduced from the absorption energy of the NPs. The oscillator strength was the only parameter varied as function of the size and shape of the embedded NP.

By a mathematical addition of the Cauchy and the Lorentz laws, we noticed that the two constants (*n*_∞_ from the Cauchy model and ε_∞_ from the Lorentz model) are only contained in the real part of the dielectric constant and correspond to an offset.

Nanospheres of 10 nm diameter were included in a 55,000 g·mol^−1^ PVP layer on a silicon substrate. Spectrophotometric measurements indicated that these nanospheres in PVP absorbed at 405 nm, i.e., the oscillator in the Lorentz model was centered at an energy of 3.06 eV. The thickness of the thin film layer, determined by a mechanical stylus profilometer (Brucker Decktak XT), was 190 ± 5 nm. The fitting parameters are summarized in Table 1.

Nanoprisms of 25 nm edge size were deposited into a 55,000 g·mol^−1^ PVP layer. Their absorption in the PVP layer was centered at 650 nm or 1.9 eV. The thin film layer thickness was 230 ± 5 nm. To fit the data, the Cauchy parameter *B* was varied. The introduction of the silver NPs reduces the amount of PVP, therefore the value of *B* was affected in comparison with the fit parameters of the PVP layer alone. The parameters of the Lorentz law, *E*_0_ and Γ, were fixed by the knowledge of the dipolar absorption peak wavelength of the corresponding NPs in PVP. The strength of the oscillator was varied in function of the NP density in the thin film layer.

Surprisingly, the nanoprisms were modeled with a single Lorentz law, accounting for the dipolar plasmonic resonance at 700 nm. Adding a second Lorentz law, accounting for quadrupolar resonances of the prisms, did not improve the fitting. The second law increases the number of variables from six to nine, but the RMSE did not decrease and the correlation coefficient *R*^2^ did not increase. This leads to the question whether the shape has an influence on the optical indices of the heterogeneous layers or only the size. According to the above samples, only the size has an influence and a single Lorentz law models the plasmonics signature of the NPs.

**Figure 3:**
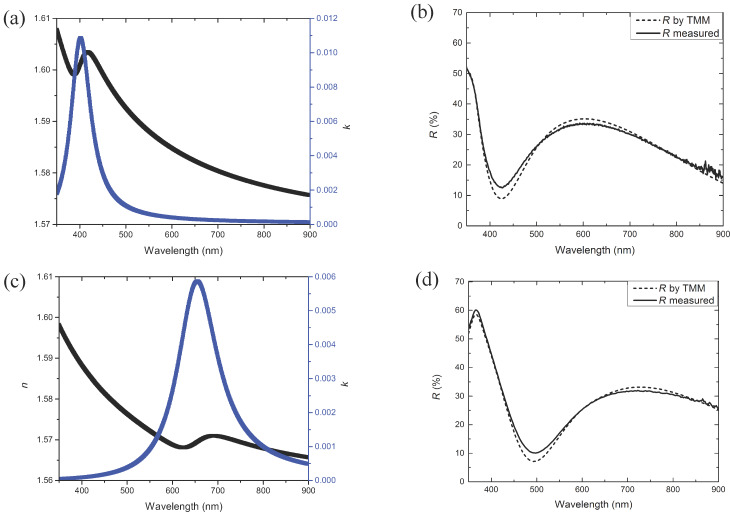
(a) Optical indices *n* and *k*, (b) reflectance measured and calculated by TMM for heterogeneous layers with nanospheres, (c) optical indices and (d) reflectance measured and calculated for heterogeneous layers with nanoprisms.

The fit to the data for the nanospheres in PVP was optimized up to a *R*^2^ = 99.45% and a RMSE = 2.1 and up to *R*^2^ = 99.44% and RMSE = 1.9 for the nanoprisms in PVP. The obtained optical indices were then used to calculate the reflectance using a TMM calculation. The calculated reflectance was compared to the measured reflectance (Figure 3) in order to validate the mathematical laws used to model the thin film layer.

With the fit quality optimized, the comparison between the measured and calculated reflectance is necessary to verify the physical validity of the fit. The two curves follow the same tendency. The slight discrepancy in the reflectance spectra could be attributed to the measurement or to diffusion phenomena induced by the NPs, which is not taken into account in our ellipsometry model.

In summary, the complex optical index of silver nanospheres and nanoprisms embedded into a transparent, non-absorbing polymer layer were modeled by the mathematical addition of the Cauchy law, accounting for the optical properties of the polymer, and the Lorentz law, centered at the dipolar absorption wavelength of the embedded NPs. The addition of further Lorentz law accounting for higher order resonances or intraband transitions occurring in the UV did not improve the data fit for this specific density of nanoprisms. Further Lorentz laws might be necessary to account for other absorption peaks when the density of nanoparticles in the thin film layer is increased.

## Conclusion

In conclusion, chemically synthesized nanospheres and nanoprisms were embedded in a transparent, non-absorbing PVP layer. Spectroscopic ellipsometry measurement results were fit in the scope of an effective medium theory with the mathematical addition of the Cauchy law and a single Lorentz law. This model is based on a multiple oscillator approach, where the effective dielectric function is the simple addition of the dielectric function of one or more Lorentz oscillators. Since the nanoparticles are randomly distributed in the layer, the effective dielectric constant accounts for the averaged optical response of the nanoprisms. A single Lorentz oscillator is required to fit the nanospheres and nanoprisms in PVP. This easy method allows us to obtain the optical indices of thin films with complex inclusions. The question arising from these results is whether the shape of the NP matters when embedded in a thin film layer. Further studies with additional shapes of NPs will be required to generalize our results, that is, the determination of whether only the size matters. The precise knowledge of the morphology of our structures, through AFM or TEM studies, may improve our optical models in the future.

## Experimental

### Synthesis of nanoparticles

**Figure 4:**
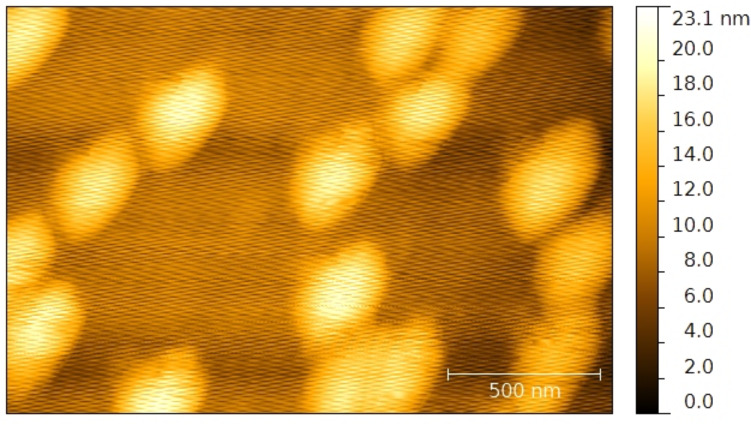
AFM topography of the nanospheres on a substrate.

The NPs were synthesized by the reduction of silver ions by sodium borohydride at room temperature in water. The nanospheres were synthesized in a one-step method [17]. The nanoprisms were created in a two-step seed-based method [18]. By depositing the nanospheres directly onto the substrate, their size was determined by atomic force microscopy (AFM, Figure 4).

The synthesized nanospheres have a diameter of 20 nm. The nanoprisms were equilateral and their edge size varied from 10 to 100 nm depending on the quantity of seed taken in the second step of the synthesis [18,37]. Their thickness was constant and found to be 10 nm. The NPs were stabilized by citrates and PVP. The colloidal solutions were washed by successive centrifugation steps and redispersed in ethanol.

### Host matrix

PVP was chosen as the host matrix due to its transparent and non-absorbing properties in the visible range. Furthermore, the pyrridil group has strong affinity to metals, such as silver NPs [28]. A few microliters of a highly concentrated PVP solution (40 g/L) in ethanol, was added to the washed NPs.

### Deposition of thin film layers

The substrates were chosen according to the measurements performed afterwards. Indeed, spectrophotometer measurements require a transparent substrate, therefore microscopic glass slides (VWR international) were used. Spectroscopic ellipsometry required a high optical index difference between the substrate and the thin film layer, thus a silicon wafer was chosen. The substrates were cleaned in an ultrasonic bath in acetone and ethanol, dried by nitrogen flow and an oxygen plasma. The latter step also increased the wettability of the substrate.

The deposition of the thin film layers was performed by spin coating. The spin coating speed was set to 500–1000 rpm, in order to obtain layers of a few hundred micrometers thickness. The deposition technique yields homogeneous thin film layers in the center of the sample where the optical measurements are performed. Nevertheless, every measurement was repeated at least twice on two different areas to avoid thickness-dependent artefacts. The thickness and homogeneity of the thin films were determined by a mechanical stylus profilometer (Brucker Decktak XT).
